# LINGO-1 shRNA protects the brain against ischemia/reperfusion injury by inhibiting the activation of NF-κB and JAK2/STAT3

**DOI:** 10.1007/s13577-021-00527-x

**Published:** 2021-04-08

**Authors:** Jiaying Zhu, Zhu Zhu, Yipin Ren, Yukang Dong, Yaqi Li, Xiulin Yang

**Affiliations:** 1 Department of Emergency, Guizhou Provincial People’s Hospital, Guiyang, 550002 Guizhou China; 2 Department of Emergency, Affiliated Hospital of Guizhou Medical University, Guiyang, 550025 Guizhou China

**Keywords:** LINGO-1, Brain, Ischemia/reperfusion, NF-κB, JAK2/STAT3

## Abstract

LINGO-1 may be involved in the pathogenesis of cerebral ischemia. However, its biological function and underlying molecular mechanism in cerebral ischemia remain to be further defined. In our study, middle cerebral artery occlusion/reperfusion (MACO/R) mice model and HT22 cell oxygen–glucose deprivation/reperfusion (OGD/R) were established to simulate the pathological process of cerebral ischemia in vivo and in vitro and to detect the relevant mechanism. We found that LINGO-1 mRNA and protein were upregulated in mice and cell models. Down-regulation LINGO-1 improved the neurological symptoms and reduced pathological changes and the infarct size of the mice after MACO/R. In addition, LINGO-1 interference alleviated apoptosis and promoted cell proliferation in HT22 of OGD/R. Moreover, down-regulation of LINGO-1 proved to inhibit nuclear translocation of p-NF-κB and reduce the expression level of p-JAK2 and p-STAT3. In conclusion, our data suggest that shLINGO-1 attenuated ischemic injury by negatively regulating NF-KB and JAK2/STAT3 pathways, highlighting a novel therapeutic target for ischemic stroke.

## Introduction

Cerebral infarction (also known as a stroke) is a common and frequently occurring disease, with high morbidity, high disability, and high mortality. Ischemia–reperfusion (IR) injury of the whole brain involves a complicated pathophysiology mechanism, and the mechanism is still not very clear [1, 2]. Reliable for infarct production, focal cerebral ischemia models, such as transient or permanent middle cerebral artery occlusion (MCAO), are the main clinical manifestation of cerebral ischemia and closely resemble a human ischemic stroke. There is no specific therapeutic drug for cerebral IR injury. However, tissue plasminogen activator (tPA) is an FDA-approved, gold standard treatment for ischemic strokes. The side effects of the treatment are to enhance the inflammatory response of the brain capillaries and nerve cell damage after stroke [3, 4]. Therefore, it is urgent to develop novel therapeutic agents and explore the underlying mechanisms of ischemic stroke.

LINGO-1 (leucine-rich repeat- and Ig-containing Nogo receptor-interacting protein-1) is a cell-surface glycoprotein selectively expressed on CNS neurons and oligodendrocytes [5]. LINGO-1 expression regulates the timing of central nervous system (CNS) myelination during development, and LINGO-1 upregulation in neurological disorders suggests a deleterious role for the endogenous protein [5]. A previous study showed that LINGO-1 might be involved in the pathogenesis of cerebral ischemia [6]. However, the molecular mechanisms of LINGO-1 engaged in the recovery of nerves after cerebral ischemia still need to be explored. Therefore, we designed this study to determine the effects of LINGO-1 on cerebral I/R injury in vivo ischemic stroke model and in vitro ischemic stroke model.

## Materials and methods

### Animals and groups

Healthy adult male C57BL/6 mice weighing 20–25 g were obtained from Beijing Experimental Animal Center and kept at 25 ± 3 °C in a room equipped with a 12-h:12-h light/dark cycle switch. Mice were randomly divided into 4 groups, including sham, MCAO/R (7 days), MCAO/R + NC, MCAO/R + sh-LINGO-1 groups. The experimental protocol was approved by the Institutional Animal Care and Use Committee of Guizhou Provincial People's Hospital (2019103) and carried out following the guidelines for the Care and Use of Laboratory Animals.

### Establishment of MCAO/R model

The modified Zea Longa suture method was conducted to establish a focal ischemia model of the middle cerebral artery occlusion (MCAO) in mice as reported previously [7]. After 2 h of focal ischemia of the middle cerebral artery, the suture plug was removed. One day, 7 days, and 14 days after MCAO/R, the mice were finally euthanized via a cardiac incision under deep anesthesia using isoflurane.

### Neurological deficit score and TTC assay

The score of the neurological deficit was executed after MCAO/R in all the mice using the modified Bederson’s method [8]. After neurological symptoms were assessed, the head was removed quickly, and the brain carefully dissected and sliced coronally at 2 mm spacing. All slices of brain tissue were stained with 1% 2,3,5 triphenyl tetrazolium chloride (TTC) solution at room temperature. The images of each staining section were taken, and the infarct area was determined using imaging analysis software(ImageJ). Normal brain tissue is bright red, and ischemic brain tissue is white. The volume percentage was expressed as the percentage of the ipsilateral hemisphere or the corrected infarct volume (mm^3^). The infarct volume was corrected for edema as previously described [9].

### Nissl staining

Paraffin-embedded tissues were sectioned and dewaxed, stained with Nissl (cresyl violet) at 37 °C, washed with PBS 2 times, and washed with 95% ethanol; then dehydrated with fresh 95% ethanol 2 times and made transparent by washing with xylene 2 times. After sealing, the Nissl bodies were observed under a light microscope.

### TUNEL assay and immunohistochemical (IHC)

TUNEL staining was performed using a TUNEL Apoptosis Assay Kit (Solarbio) as described in our previous study [10]. The TUNEL staining was performed according to the manufacturer’s protocol. Finally, a light microscope and LEICA software version 2.0 was used to analyze TUNEL staining. For the mouse brain, a standard procedure was utilized for IHC staining on 4-μm sections prepared from paraffin-embedded tissue samples of ischaemic brains. Finally, immunoreactivity was visualized with diaminobenzidine. A brown colour staining was considered a positive result.

### Cell culture and OGD/R treatment

The mouse hippocampal neuron cell line HT22 cells were obtained from Shanghai Cell Bank of the Chinese Academy of Sciences (Shanghai, China), cultured in DMEM medium, supplemented with 10% FBS (Hyclone), 100 U/ml penicillin, and 100 μg/ml streptomycin as described previously [11] in a humidified 5% CO_2_ incubator at 37 °C. For the OGD treatment, cells were transferred to a hypoxic chamber with 95% N_2_ and 5% CO_2_ and cultured in DMEM medium without glucose and FBS at 37 °C. After 12 h/24 h/48 h OGD treatment, the medium of the cells was changed with a normal medium, and the cells were returned to the normal incubator to mimic reperfusion.

### Proliferation assay

Cell viability and cytotoxicity assay were measured with CCK8 Kit (Solarbio) according to the instructions of the manufacturers. A defined number of cells were seeded at a density of 1 × 10^4^/well in a complete growth medium in 96-well plates. The plates were incubated at 37 °C for 1.5 h and then analyzed in a multiwall plate reader (BioTek Instruments, Inc.).

### Lentiviral infection

A LINGO-1 shRNA lentiviral vector was synthesized by HANBIO Company and infection was performed according to the manufacturer’s manual.

### Immunofluorescence (IHC) staining analysis

Cells were fixed with 4% paraformaldehyde (Sigma) and blocked with 5% bovine serum albumin for 30 min. Primary antibodies in the blocking solution were added, and cells were incubated overnight at 4 °C. Subsequently, the cells were incubated with fluorogenic secondary antibodies for 2 h at room temperature and then incubated with DAPI. Sections were imaged with a laser confocal microscope (Nikon, Japan).

### Real-time quantified PCR (qRT-PCR)

Total RNA was isolated with TRIZOL reagent (Invitrogen, USA) according to the manufacturer’s instructions and subjected to reverse transcription reactions using PrimeScript RT reagent kit (Takara, China). Samples were analyzed on an ABI 7300 qPCR instrument (Applied Biosystems, USA).

### Western blot assay

Protein was extracted from samples (tissue and cultured cells) in RIPA buffer contained 1 mM PMSF (SolarBio, China) and separated on 8–10% SDS-PAGE. Then the proteins were transferred to polyvinylidene fluoride (PVDF) membrane (Millipore, USA) with being probed with LINGO-1 (1:1000; ab23631; Abcam), GAPDH(1:5000; ab8245; Abcam), Bax (1:1000; ab182734; Abcam), Bcl-2 (1:1000; ab194583; Abcam), Caspase 3 (1:1000; ab184787; Abcam), β-Actin (1:5000; ab8227; Abcam), NF-κB (1:1000; ab16502; Abcam), p-NF-κB (1:1000; ab194726; Abcam), JAK2 (1:1000; ab108596; Abcam), p-JAK2 (1:1000; ab32101; Abcam), STAT3 (1:1000; ab68153; Abcam) and p-STAT3 (1:1000; ab76351; Abcam) for 24 h at 4 °C. Afterward, the proteins were incubated with appropriate secondary antibody for 2 h at about 25 °C. The density of the immunoreactive bands was quantified using ImageJ software.

### Statistical analysis

For in vitro experiments, results are expressed as the mean ± SD, and for in vivo experiments, results are expressed as the mean ± SEM. Means of two groups were compared using independent-samples *t* tests, and means of multiple groups were compared by one-way analyses of variance. A value of *p* < 0.05 was considered statistically significant.

## Results

### LINGO-1 expression is up-regulated in ischemia/reperfusion brain injury mice or cell model

**Fig. 1:**
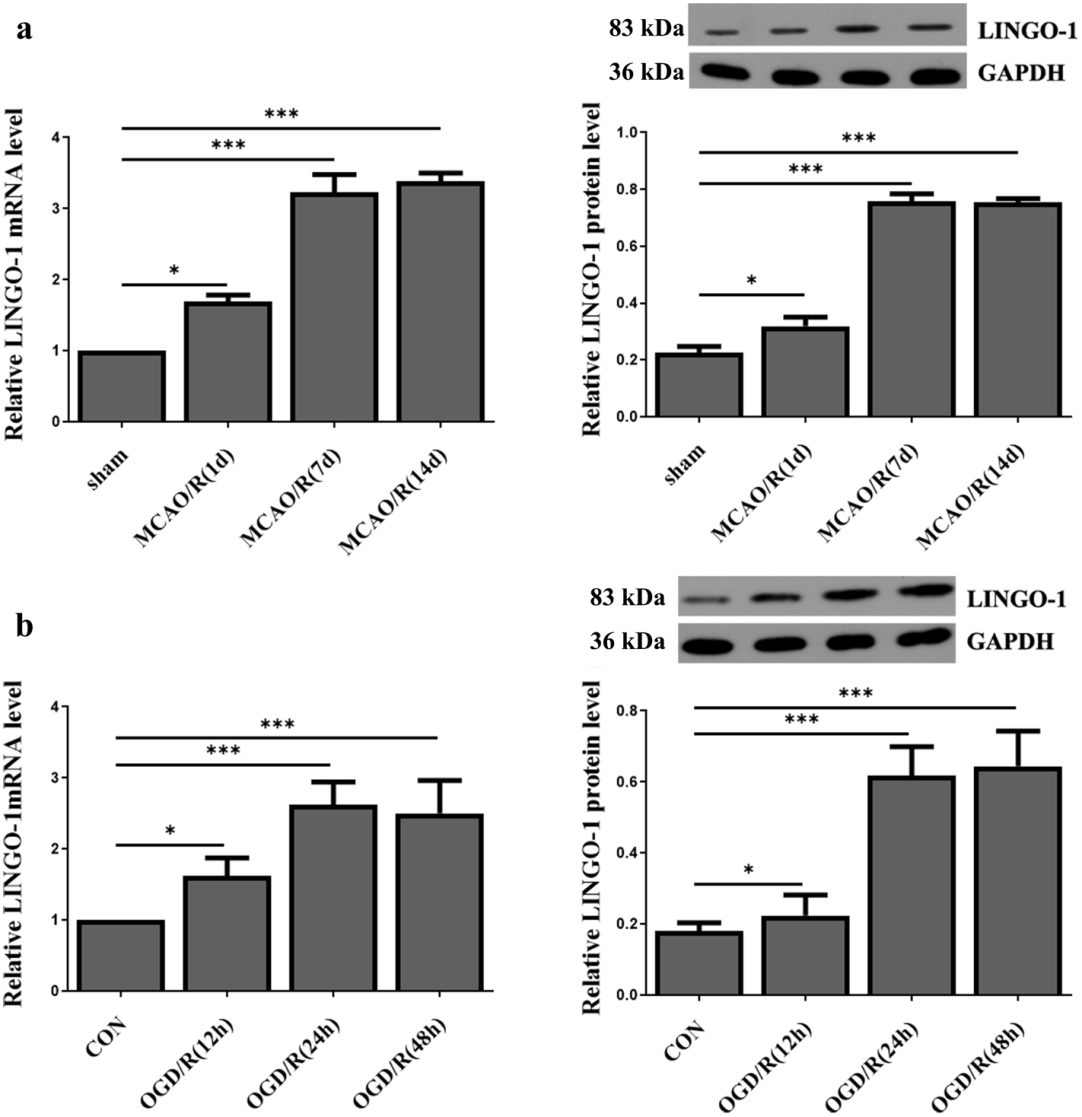
LINGO-1 was overexpression in MACO/R and OGD/R model. **a** Study design and timeline of experiments in vivo and in vitro. **a** Relative LINGO-1 mRNA and protein expression in MACO models 1, 7, and 14 days of reperfusion. Data are expressed as mean ± SEM. **b** Relative LINGO-1 mRNA and protein expression in HT22 cell models 12, 24, and 48 h of reperfusion. Data are expressed as mean ± SD. and *p < 0.05; **p < 0.01; ***p < 0.001

MACO/R mice model and OGD/R cell model were established to determine whether LINGO-1 was involved in the brain I/R injury. We determined the expression level of LINGO-1 in cell and mice models using qRT-PCR and WB. We found that LINGO-1 was aberrantly up-regulated in the MACO/R model after 1, 7, and 14 days of reperfusion compared to that in the sham group (Fig. 1a). LINGO-1 began to increase at 1 day after MACO/R, peaked at 14 days, and slightly decreased at 21 days. The mRNA and protein expression levels of LINGO-1 were also detected in the OGD/R cell model. (Fig. 1b).

### Down-regulation of LINGO-1 reduce ischemia/reperfusion brain injury in mice

**Fig. 2:**
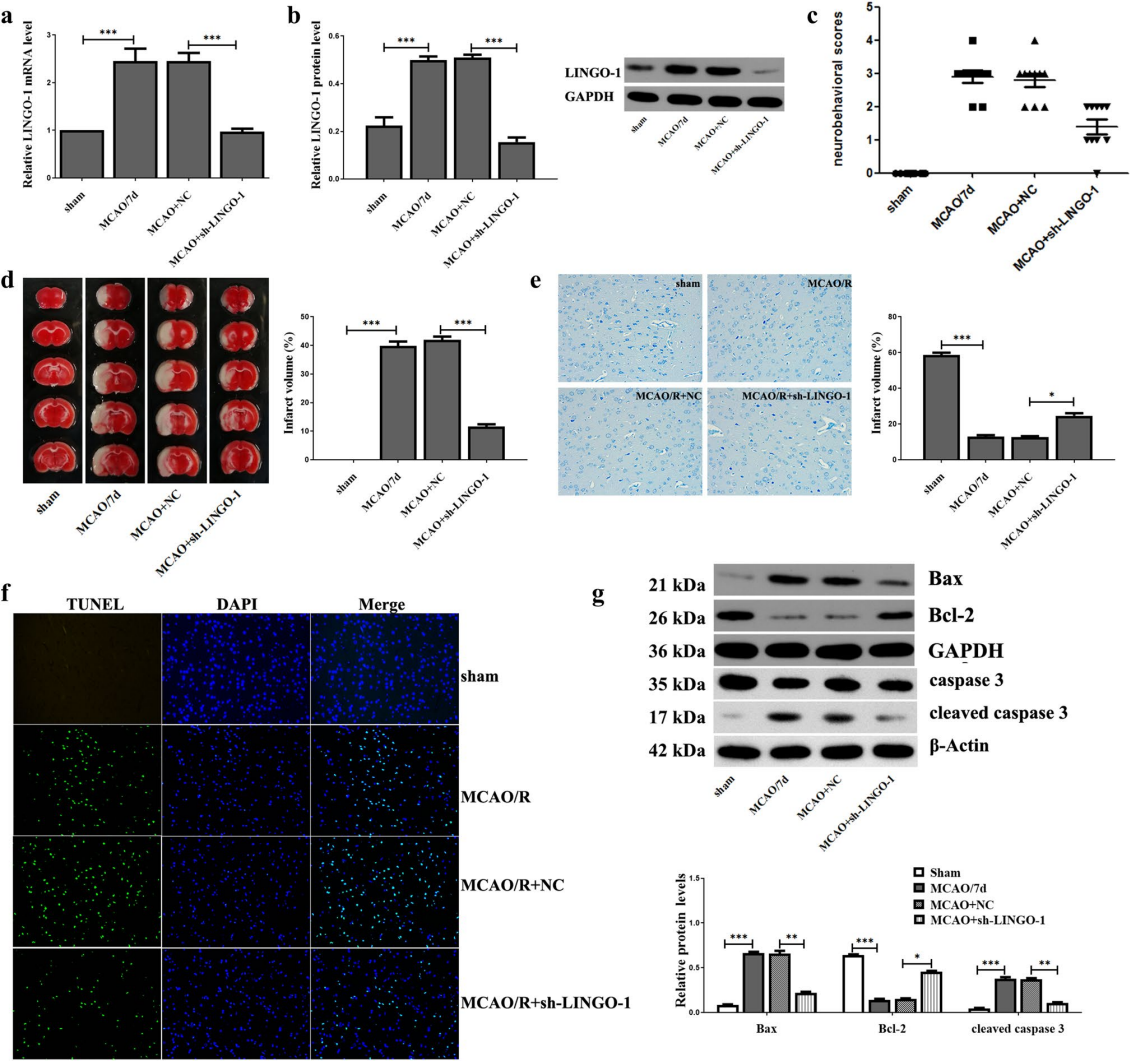
Effect of sh-LINGO-1 on I/R-induced MACO mice model. **a**, **b** LINGO-1 mRNA and protein expression were detected by qRT-PCR and WB at 7 days after MCAO. **c** Neurobehavioral score determination (Longa scoring system) Sham, MCAO/R (7 days), MCAO/R + NC, MCAO/R + sh-LINGO-1 (lentiviral vectors) groups. **d** Brain infarct region in TTC staining. Red: Non-ischemic area; white: ischemic area. Percentage of infarct volume after TTC staining. **e** Nissl staining. **f** TUNEL staining. **g** Expression of apoptosis-related proteins Bax, Bcl-2, Caspase 3, and Cleaved caspase 3 were detected by WB. Data are expressed as mean ± SEM and *p < 0.05; **p < 0.01; ***p < 0.001

Interference fragments of LINGO-1 (sh-LINGO-1, MACO/R mice injected with sh-LINGO-1 plasmid) were used to investigate the effect of LINGO-1 in the MACO mice model. In the MACO/R model, the results of qRT-PCR and WB showed that compared with the negative control (MCAO/R + NC), the sh-LINGO-1 group showed markedly reduced expression of LINGO-1 (Fig. 2a, b). Compared with the sham group, neurobehavioral scores were significantly increased in the MACO/7d group but significantly reduced in the MCAO/R + sh-LINGO-1 group (Fig. 2c). TTC staining showed obvious infarcts in the brain tissue of the MCAO/R group. However, treatment with a sh-LINGO-1 induced a less severe infarct than the MCAO group and led to a significant decrease in the infarct area compared with MCAO (Fig. 2d). Nissl staining showed that the number of neurons with normal morphology in the hippocampus of the MCAO/R + sh-LINGO-1 group was higher, and the neuron gap was reduced compared with that of the MCAO/R group. (Fig. 2e). Moreover, the TUNEL analysis revealed that inhibiting LINGO-1 could significantly decrease the rate of apoptosis in MCAO (Fig. 2f). Consistently, knockdown of LINGO-1 significantly decreased the level of Cleaved caspase 3 and Bax protein, increased Bal-2 compared with MCAO/R group (Fig. 2g). Together, these data indicate that the sh-LINGO-1 is effectively protected against brain tissue damage in the MCAO/R model.

### Down-regulation of LINGO-1 increased cell viability and decreased apoptosis in OGD-induced HT22 cells

**Fig. 3:**
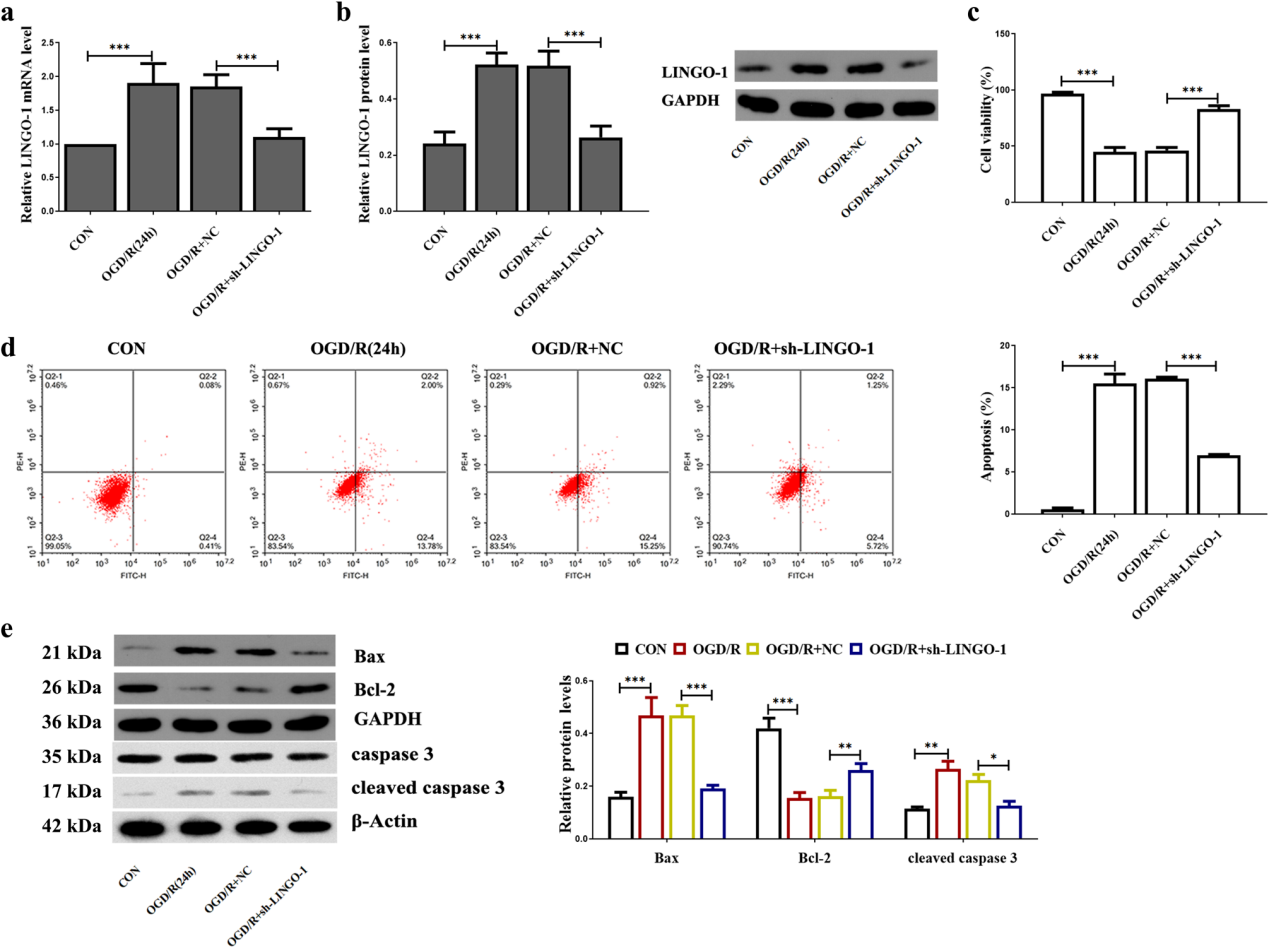
Down-regulation of LINGO-1 increased cell viability and decreased apoptosis in OGD-induced HT22 cells. **a** LINGO-1 mRNA expression was detected by qPCR, and **b** WB was used to assay LINGO-1 protein expression. **b** Cell viability of HT22 cells during OGD/R treated with sh-LINGO-1 was measured by CCK8. **c** Apoptosis was examined by flow cytometric analysis using Annexin V/PI assay. **d** Expression of apoptosis-related proteins Bax, Bcl-2, Caspase 3, and Cleaved caspase 3 were detected by western blot. Data are expressed as mean ± SD and *p < 0.05; **p < 0.01; ***p < 0.001

To verify the efficiency of knockdown LINGO-1 in HT22 cells, we used RT-PCR and WB to detect the mRNA and protein levels. As shown in Fig. 3a, b, LINGO-1 in the OGD/R + sh-LINGO-1 group was significantly lower than that in the OGD/R group. Figure 3c shows that cell viability was decreased in a time-dependent manner during OGD and reperfusion. OGD for 12 h and reperfusion for 24 h cell viability were reduced to about 50% compared with normal cells. Treatment of sh-LINGO-1 increased cell viability. Results of flow cytometry (Fig. 3d) showed that inhibiting LINGO-1 could partially reverse the OGD/R-induced apoptosis in HT22 cells. Next, we detected the apoptosis-related protein level. Figure 3f shows that transfection of sh-LINGO-1 significantly reduces the high expression of apoptotic proteins Cleaved caspase 3 and Bax induced by I/R injury. The expression trend of anti-apoptotic protein Bcl-2 was contrary to that of Cleaved caspase 3 and Bax.

### Down-regulation of LINGO-1 inhibited the activation of NF-KB and JAK2/STAT3 both in vivo and in vitro ischemic stroke models

**Fig. 4:**
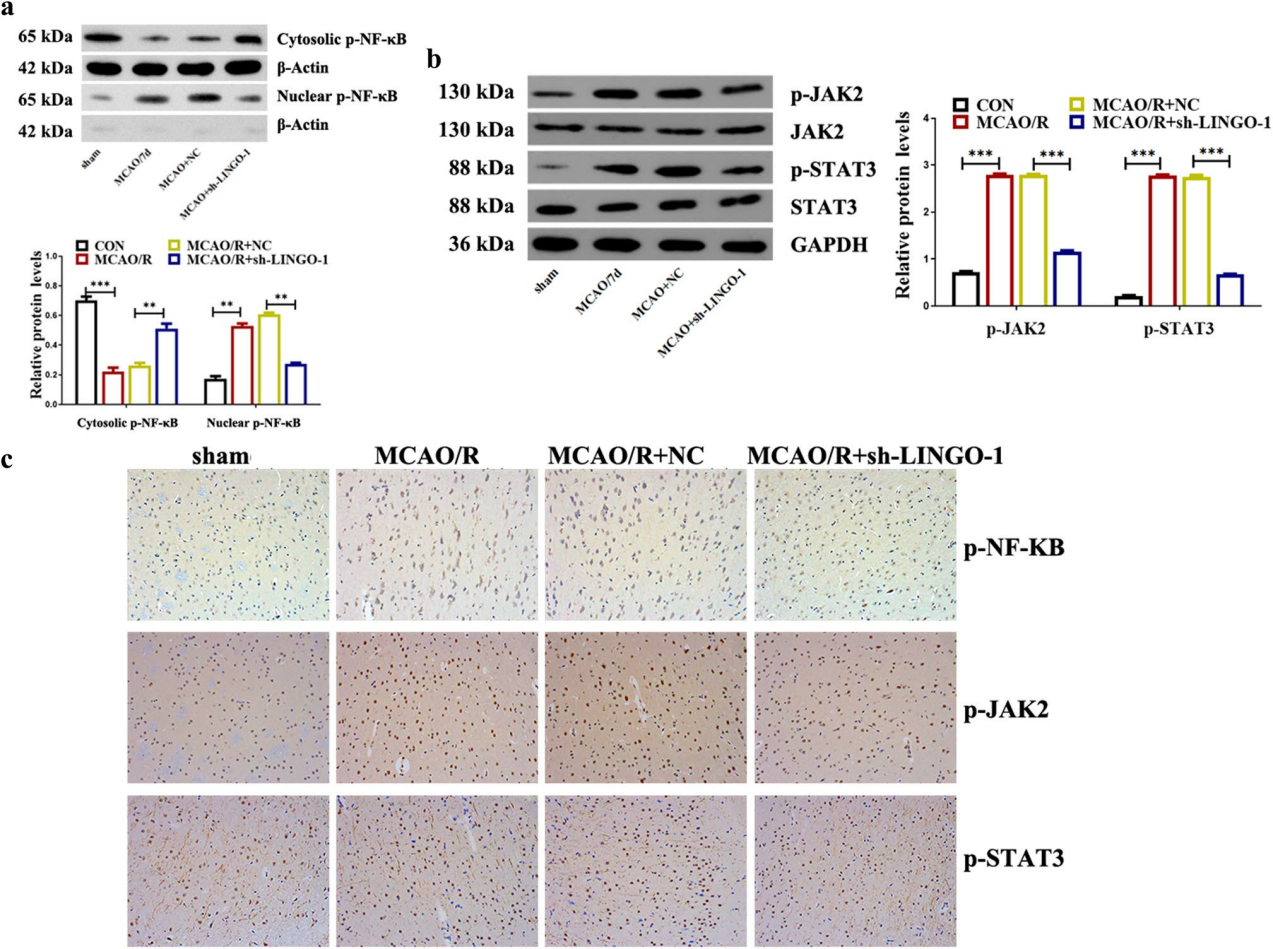
Knockdown LINGO-1 inhibited the activation of NF-kB and STAT3 in the MACO/R mice model. **a** Protein levels of p-NF-κB in the cytoplasm and nuclear. **b** The protein levels of p-JAK2, JAK2 p-STAT3, and STAT3 in different groups were detected by WB and quantified by scanning densitometry, and the ratio phospho/total was represented. **b** IHC was used to detect the expression of p-NF-κB, p-JAK2, and p-STAT3 proteins. Data are expressed as mean ± SEM and *p < 0.05; **p < 0.01; ***p < 0.001

As shown in Fig. 4a, the WB data using cytoplasmic and nuclear extracts indicated that inhibition of LINGO-1 significantly suppressed the nuclear translocation of p-NF-κB, as compared with the MCAO/R group. Moreover, the WB results showed decreased levels of p-JAK2 and p-STAT3 in tissue extracts from the ischemic cortex in the MCAO/R + sh-LINGO-1 group compared with the MCAO/R group (Fig. 4b). The immunoreactivity of p-NF-κB, p-JAK2, and p-STAT3 was significantly decreased in the MCAO/R + sh-LINGO-1 group compared with the MCAO/R group (Fig. 4c).

**Fig. 5:**
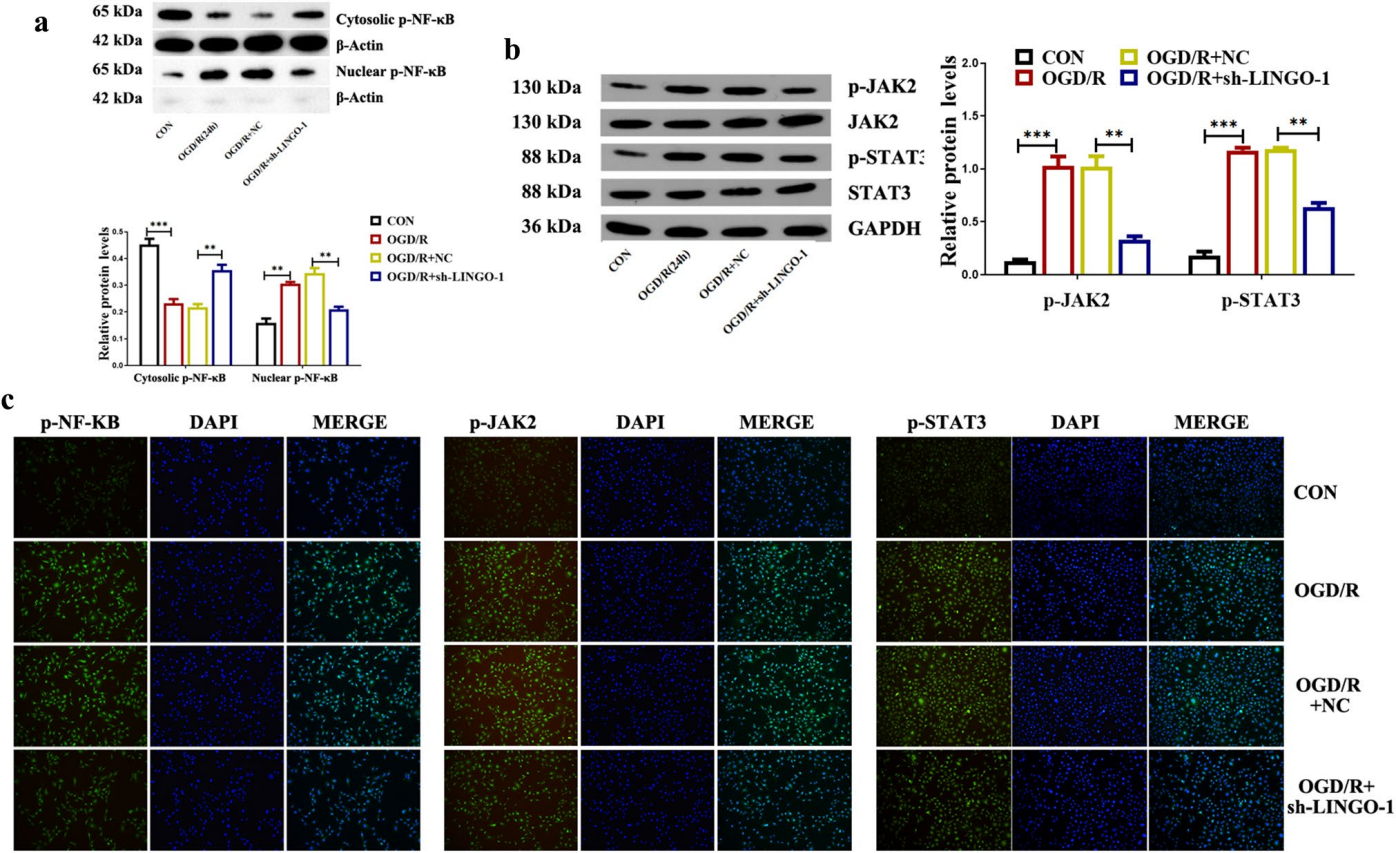
Knockdown LINGO-1 inhibited the activation of NF-kB and STAT3 in the OGD/R cell model. **a** Protein levels of p-NF-κB in the cytoplasm and nuclear. **b** The protein levels of p-JAK2, JAK2 p-STAT3, and STAT3 in different groups detected by WB and quantified by scanning densitometry, and the ratio phospho/total was represented. **b** IF was used to detect the expression of p-NF-κB, p-JAK2 and p-STAT3 proteins. Data are expressed as mean ± SD and *p < 0.05; **p < 0.01; ***p < 0.001

Besides, HT22 cells were transfected with sh-LINGO-1 to verify the effect of LINGO-1 on the NF-κB and JAK2/STAT3 signaling pathway. As shown in Fig. 5a, p-NF-κB was translocated into the nucleus in OGD/R-induced HT22 cells. However, transfection with sh-LINGO-1 significantly suppressed the nuclear translocation of p-NF-κB in OGD/R-induced HT22 cells. Besides, the findings of WB and IF showed that p-JAK2, p-STAT3, and p-NF-κB were markedly decreased by transfection with sh-LINGO-1 compared with the OGD/R group, suggesting that LINGO-1 knockdown induced inhibition on the NF-KB and JAK2/STAT3 pathway (Fig. 5b, c).

## Discussion

**Fig. 6:**
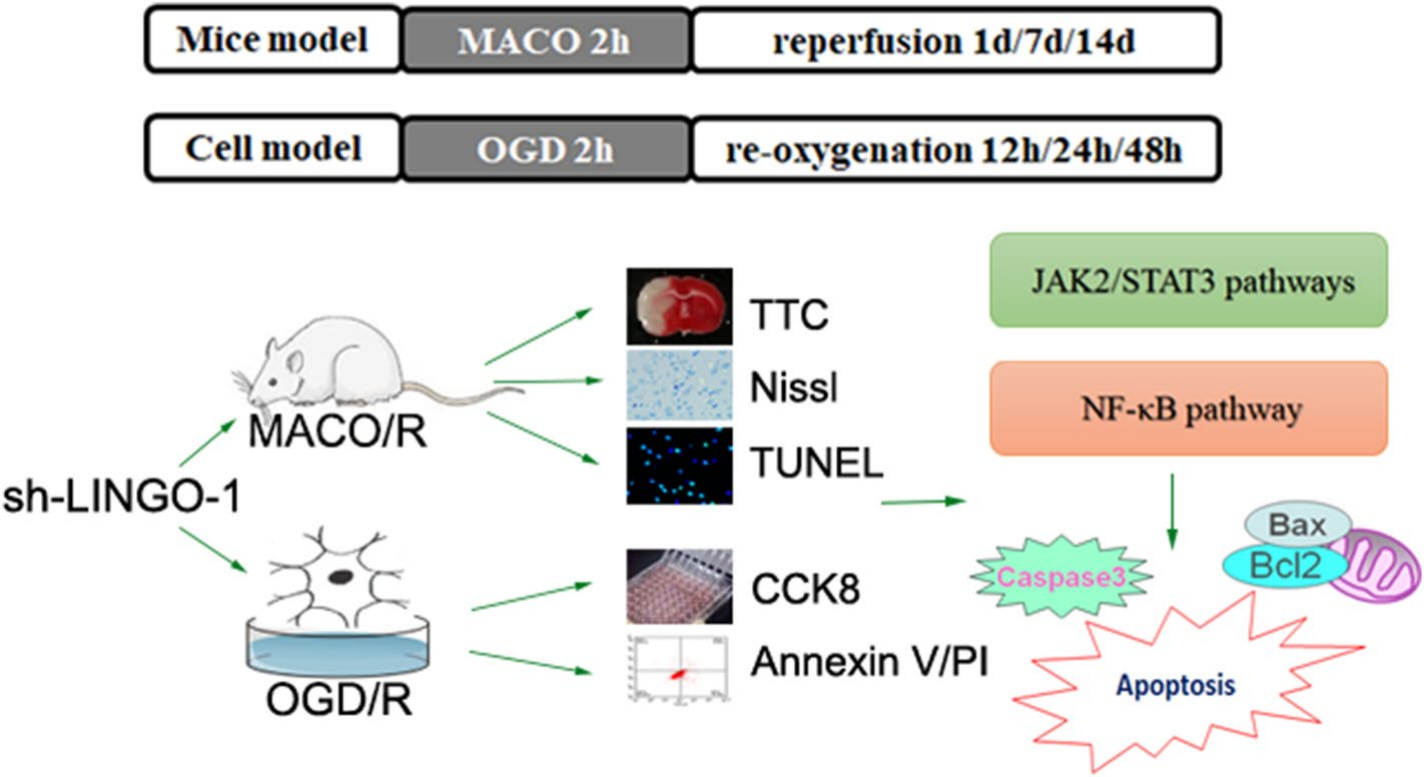
The neuroprotective effect of sh-LINGO-1 in both in vivo and in vitro ischemic stroke models

Cerebral ischemia is a major cause of brain injury, and its management remains challenging [12]. In the process of cerebral I/R, the imbalance of neuronal DNA damage-repair mechanism can cause the irreversible damage of neuronal DNA, and then lead to the modulation and death of neurons [13]. In our present study, we demonstrate that the neuroprotective effect of sh-LINGO-1 in both in vivo and in vitro ischemic stroke models (Fig. 6).

LINGO-1 is a transmembrane signaling protein, mainly expressed in the brain, notably within both the cerebral cortex and the limbic system [14, 15]. Ischemic brain injury is a multifactor and multilevel complex pathological process based on nerve injury. One of the main reasons that axons cannot regenerate after nervous system injury is that the myelin sheath produces nerve growth-inhibiting factors. Nogo receptor (NgR) complex, a kind of CNS myelin protein, played a key role in inhibiting neurite growth. LINGO-1 is an important member of the NgR complex that transmits signals of myelin inhibition [16–19]. A previous study found that transplantation of LINGO-1-RNA interference-treated neural stem cells facilitates functional recovery after spinal cord injury by inactivated the RhoA and Notch signaling pathways [20]. A recent study showed that LINGO-1 deficiency promotes nerve regeneration through the reduction of cell apoptosis, inflammation, and glial scar after SCI in mice [5]. So far, it is unclear the complicated molecular mechanism of LINGO-1 after cerebral ischemia. This study proved that LINGO-1 was overexpressed in the mouse brain after MCAO and in HT22 cells after OGD/R, which may be one reason for the nerve regeneration disorder after cerebral ischemia. Blocking LINGO-1 function leads to ameliorates neurological deficits, reduces brain infarct volume and damage to cerebral cortical neurons after MCAO/R in mice. Subsequently, we revealed that knockdown of LINGO-1 could promote cell proliferation and decreased apoptosis in HT22 following OGD/R. These data indicated that LINGO-1 shRNA might have a protective effect in cerebral ischemia/reperfusion injury.

Emerging evidence suggests that neuronal cell apoptosis is a predominant pathological issue in numerous neurological disorders, including cerebral I/R injury [21]. According to TTC staining, sh-LINGO-1 treatment reduced the infarct area compared with the MCAO group, indicating that sh-LINGO-1 may play a protective role in MCAO. A previous study showed that the TUNEL assay showed that inhibition of LINGO-1 could significantly reduce apoptosis compared with the MCAO/R group, and down-regulate the expression of Bax and Cleaved caspase 3, up-regulate the expression of Bcl-2, suggesting that LINGO-1 may block the apoptosis pathway. The Bcl-2 family proteins may play a critical role in ischemic neuronal death. Also, ischemia increased Cleaved caspase 3 activities, and caspases may act as treatment targets in stroke and neurodegenerative diseases.

Ischemic brain injury involves the disturbance of stable networks involving multiple signaling pathways and a variety of genes. Among them, NF-κB has been considered as a prominent controller of a variety of pathological or cell death cascades [22]. Additionally, JAK2 and STAT3 phosphorylation are elevated in ischemia–reperfusion and adriamycin-induced nephropathy [23, 24]. Satriotomo et al. showed that blockage of STAT3 phosphorylation with either a JAK2 inhibitor or STAT3 siRNA significantly decreased infarction, apoptosis, and neurological dysfunction in the post-ischemic brain [25]. Here, the present study revealed that LINGO-1 shRNA treatment suppressed the activation of the JAK2/STAT3 and NF-κB signaling pathways in both vivo and in vitro ischemic stroke models.

In conclusion, the results of this study indicate that LINGO-1 shRNA could provide neuroprotection against I/R injury. LINGO-1 shRNA could ameliorate neurological deficits, reduce brain infarct volume and damage to cerebral cortical neurons in vivo ischemic stroke model, alleviate apoptosis and promote cell proliferation in vitro ischemic stroke model via the NF-κB and JAK2/STAT3 pathway.
